# Third-stage *Gnathostoma spinigerum* larva excretory secretory antigens modulate function of Fc gamma receptor I-mediated monocytes in peripheral blood mononuclear cell culture

**DOI:** 10.1186/s41182-016-0005-x

**Published:** 2016-04-21

**Authors:** Surachet Benjathummarak, Ratchanok Kumsiri, Supaporn Nuamtanong, Thareerat Kalambaheti, Jitra Waikagul, Nareerat Viseshakul, Yaowapa Maneerat

**Affiliations:** Center of Excellence for Antibody Research, Faculty of Tropical Medicine, Mahidol University, 10400 Bangkok, Thailand; Pathobiology Unit, Department of Medical Science, Faculty of Science, Rangsit University, Pathumthani, 12000 Thailand; Department of Helminthology, Faculty of Tropical Medicine, Mahidol University, Bangkok, 10400 Thailand; Department of Microbiology and Immunology, Faculty of Tropical Medicine, Mahidol University, Bangkok, 10400 Thailand; Parasitology Unit, Department of Pathology, Faculty of Veterinary Science, Chulalongkorn University, Bangkok, 10330 Thailand; Department of Tropical Pathology, Faculty of Tropical Medicine, Mahidol University, Bangkok, 10400 Thailand

**Keywords:** *Gnathostoma spinigerum*, Excretory-secretory, FcγRI, Monocytes, Phagocytosis

## Abstract

**Background:**

Third (infective)-stage *Gnathostoma spinigerum* larvae (L3) mainly cause human gnathostomiasis. *G. spinigerum* L3 migrate throughout the subcutaneous tissues, vital organs, and central nervous system and can cause various pathogenesis including sudden death. Interestingly, *G. spinigerum* L3 can survive and evade host cellular immunity for months or years. The effects of *G. spinigerum* excretory-secretory (ES) products involved in larval migration and immune-evasive strategies are unknown. Monocytes are innate immune cells that act as phagocytic and antigen-presenting cells and also play roles against helminthic infections via a complex interplay between other immune cells. Fc gamma receptor I (FcγRI) is a high-affinity receptor that is particularly expressed on monocytes, macrophages, and dendritic cells. The cross-linking of FcγRI and antigen-antibody complex initiates signal transduction cascades in phagocytosis, cytokine production, and antibody-dependent cell-mediated cytotoxicity (ADCC). This study investigated whether ES antigen (ESA) from *G. spinigerum* L3 affects monocyte functions.

**Results:**

Cultures of normal peripheral blood mononuclear cells (PBMC) separated from healthy buffy coats were used as a human immune cell model. ESA was prepared from *G. spinigerum* L3 culture. Using Real-Time quantitative reverse transcription-polymerase chain reaction (qRT-PCR), the effect of ESA to down-regulate FcγRI mRNA expression in monocytes during 90 min of observation was not well delineated. Flow cytometry analysis revealed a significant phenotypic-decreased FcγRI expression on the monocyte surface at 12 hours (h) of cultivation with the ESA (*p* = 0.033). Significantly reduced monocyte-mediated phagocytosis capacity was consistently observed after 12 h of ESA pretreatment (*p* = 0.001).

**Conclusions:**

Our results suggest that *G. spinigerum* ESA modulates monocyte function via depletion of FcγRI expression. This study provides preliminary information for future in-depth studies to elucidate mechanisms of the immune-evasive strategy of *G. spinigerum* larvae.

**Electronic supplementary material:**

The online version of this article (doi:10.1186/s41182-016-0005-x) contains supplementary material, which is available to authorized users.

## Background

Previous studies have indicated that cutaneous and visceral larva migrans are associated with excretory-secretory (ES) products from infective helminthic larvae [1, 2]. ES products are substances released by parasites during in vitro cultivation or released in vivo as exhibited by the increase of specific antibodies against various ES proteins in infected humans and animals [1]. ES protein molecules have divergent functions that contribute to different activities of the parasites. ES products include (1) hydrolytic enzymes, such as protease and hyaluronidase for digestion, tissue invasion, and degradation of host proteins for nourishment, (2) protease inhibitors for anticoagulation, (3) inhibitors of platelet activation, (4) anti-inflammatory agents, and (5) modulators of host immune responses [2–5].

Third-stage *Gnathostoma spinigerum* larvae (L3) mainly cause human gnathostomiasis. L3 cannot undergo further development into the adult form in infected patients. Instead, they continue to migrate throughout subcutaneous tissues, vital organs, and the central nervous system and can cause various forms of pathogenesis, including sudden death. Interestingly, L3 can survive and evade host cellular immunity for months or even years [6]. However, the functions and effects of the ES products involved in larval migration and immune-evasive strategies are unknown.

Fc receptors (FcR) are membrane glycoproteins with an affinity for the Fc portions of secreted antibodies. Three classes of FcγR based on genetic homology, Fc gamma receptor I (FcγRI), FcγRII, and FcγRIII, are critically involved at multiple stages of immune responses [7]. FcγR molecules can potently enhance antigen presentation, and the type of FcγR involved has been shown to be a crucial determinant for the types of epitopes presented by the antigen-presenting cell (APC) [7].

The human high-affinity receptor for IgG, FcγRI (CD64), is constitutively expressed on APC, e.g., monocytes, macrophages, neutrophils, and dendritic cells (DC). FcγRI expression is up-regulated by stimulation with cytokines, such as interferon-gamma (IFN-γ) [8] or interleukin-2 (IL-2) [9]. The cross-linking of FcγRI by binding of antigen-antibody complex causes the initiation of signal transduction cascades that result in phagocytosis, cytokine production, or antibody-dependent cell-mediated cytotoxicity (ADCC) [7, 10].

Monocytes are immune cells that play a critical role against helminthic infections via a complex interplay between antibodies and other immune cells, particularly T cells, eosinophils, basophils, and mast cells (MC). Monocytes are recruited to sites of infection and are precursors of specific macrophage and DC populations at tissue sites. Monocytes have both innate immune and subsequent APC functions in response to helminth infection. Unlike unicellular pathogens, e.g., bacteria, virus, and protozoa, helminths are too large to be digested by phagocytosis. Instead, monocytes and macrophage can internalize helminth antigens [11] and soluble substances produced by the helminths [12, 13], then process and present the antigen on their surfaces.

In the primary immune response, APCs process helminth antigens and present them to CD4+ T cells that differentiate into T helper 2 (Th2) cells. Th2 cells produce cytokines, such as IL-4, -5, 9, and -13, which stimulate and attract macrophages, eosinophils, basophils, and other innate immune cells and B cells. Moreover, IL-4 and -13 are involved in the differentiation of antigen-specific B cells and the production of large amounts of antibodies (characteristically IgE). Antibodies opsonize helminths, leading to killing via eosinophils or neutrophils, as well as by macrophages through the mechanism of ADCC. IgE binds to Fcε-receptors (FcεRI) on MCs and basophils. Consequently, sensitized MC and basophils secrete large amounts of histamine and other mediators and facilitate the attraction and accumulation of further immune cells, resulting in the killing of the helminth (reviewed in [12, 14]).

The present study aimed to investigate whether ES antigens (ESA) from *G. spinigerum* L3 affected monocyte functions. We used cultures of normal peripheral blood mononuclear cells (PBMC) separated from five healthy buffy coats as a human immune cell model. Firstly, PBMC were exposed to ESA obtained from the L3 culture to titrate the appropriate dose for this study. Secondly, we determined the effect of *G. spinigerum* ESA on the decrease of (1) FcγRI-encoded mRNA expression by Real-Time quantitative reverse transcription-polymerase chain reaction (qRT-PCR) techniques, (2) FcγRI expression on monocytes in PBMC culture using flow cytometry (FACS), and (3) monocyte-mediated phagocytosis capacity by zymosan (ZM) phagocytic assay. These novel findings explain how monocyte-mediated mechanisms might contribute to immune-evasive strategies in human gnathostomiasis.

## Methods

### Chemicals

For cell culture, we used Roswell Park Memorial Institute (RPMI) 1640 medium without phenol red (Cat. No. 118535–063) and with phenol red (Cat. No. 31800022); 6-well transwell cell culture plates from Corning, NY, USA (Cat. No. 3421); fetal bovine serum (FBS) from Gibco, Grand Island, NY, USA; and recombinant (r)IL-2 and rTGF-β1 (ProSpec-Tany TechnoGene Ltd., Rehovot, Israel). Fluorescence isothiocyanate (FITC) or PE-conjugated antibodies to CD14, CD27, CD64, and propidium iodide (PI) (BioLegend, San Diego, CA, USA) were used for immunostaining for FACS analysis. The RNA isolation kit and DNase treatment step (QIAGEN, Hilden, Germany) and Affymetrix GeneChip® Human Gene 1.0 ST arrays (Affymetrix Inc., Santa Clara, CA, USA, FITC-conjugated Zymosan A and lyticase (Sigma, Saint Louis, MO, USA)) were also utilized.

### Study design and subjects

This study was conducted at the Faculty of Tropical Medicine, Mahidol University. The study was approved by the Ethics Committees of the Faculty of Tropical Medicine, Mahidol University (MUTM2013-079-01) and the Thai Red Cross Society (Bangkok, Thailand). Normal PBMC were separated from five healthy buffy coats provided by the Thai Red Cross Society. These PBMC were used as a human immune cell model in the present study.

### PBMC

Buffy coats from O^+^ blood from healthy donors were purchased from the Thai Red Cross Society. Gradient centrifugation and lymphoprep (Axis-Sheld Poc AS, Oslo, Norway) were used to separate PBMC from buffy coat samples as previously described [15]. To limit interference from adaptive immunity, anti-human CD27 and LD columns [16] were used to remove memory B cells (CD27^+^) from each PBMC sample. Based on FACS analysis, PBMC cultures used in each experiment were comprised of less than 1 % memory B cells.

### Preparation of ESA from *G. spinigerum* L3 culture (*G. spinigerum* ESA)

*G. spinigerum* ESA was prepared [17] to verify the effects of ESA on PBMC cultures. Briefly, *G. spinigerum* L3 were cultured in RPMI 1640 medium without phenol red and with supplements containing 1 μg/ml of gentamycin. The larvae were cultured in 2 ml of medium in 6-well plates (10 larvae/well) at 37 °C in an atmosphere of 5 % CO_2_ and maintained for 1 month. Of culture medium, 1 ml was collected and replaced with an equal volume of fresh medium every 24 h. The culture medium was collected and maintained at −20 °C throughout the cultivation time. The pooled medium, approximately 300 ml, was lyophilized at −20 °C then dissolved with 0.1 M PBS to a final volume of 10 ml. Impurities in the ESA were then removed using dialyzing sacks at a MW cutoff of 12,400 Da (Cat. No. D0405, Sigma-Aldrich, Gillingham, Dorset, UK) with 0.1 M PBS overnight at 4 °C. The purified ESA was kept at −20 °C until used. A Coomassie® Plus Protein Assay Reagent Kit (Pierce, Rockford, IL, USA) and a Nanodrop ND1000 Spectrophotometer (Thermo Scientific, Wilmington, DE, USA) were used to determine the protein concentration of the purified ESA. The absence of endotoxin from the purified ESA was confirmed using Limulus amebocyte lysate test (E-TOXATE kit, Sigma-Aldrich, St. Louis, MO, USA).

### Determination of appropriate concentration of *G. spinigerum* ESA for PBMC culture

In all, 2 × 10^6^ PBMC (CD27^−^) were cultured in RPMI1640 supplemented with 10 % inactivated FBS alone or with IL-2 (10 ng/ml) [9] or ESA (0.1, 0.5, and 1 μg/ml). After incubation for 12 or 24 h, the cultured PBMC were washed in 0.1 M PBS containing 0.01 % BSA, then stained with 0.1 μg of PI for 15 min at room temperature in the dark. The intensity of PI-positive staining was then analyzed by a FACSCalibur flow cytometer and CellQuest software (Becton Dickinson, San Jose, CA, USA). The number of dead cells (PI-positive stained cells) was determined and compared with PBMC treated with different concentrations of ESA and IL-2 or in medium alone.

### Investigation of FcγRI mRNA expression

In all, 3 × 10^6^ PBMC (CD27^−^) were cultured in RPMI 1640 supplemented with 10 % FBS alone or plus IL-2 [9], TGF-β1 [18], or ESA (0.1 μg/ml). After incubation for 15, 30, 60, or 90 min [19], the cultured PBMC were harvested and immediately washed with PBS. Total RNA was extracted from the PBMC using the RNeasy total RNA isolation kit and a DNase treatment step (QIAGEN, Hilden, Germany). Then cDNA was synthesized using 1 μg of total RNA with a SuperScript III First-Strand Synthesis System for RT-PCR (Invitrogen, USA) according to the manufacturer’s protocol. Each 20 μl of PCR reaction contained 10 μl of LightCycler 480 SYBR Green I Master mix (Roche Diagnostic, Mannheim, Germany) and was mixed with 100 ng of cDNA and a specific primer (1 μM) in a LightCycler 480 instrument. The FcγRI gene was amplified with the following primers: FcγRI 5′-GTGTCATGCGTGGAAGGATA-3′ (forward) and FcγRI 5′-GCACTGGAGCTGGAAATAGC-3′ (reverse) (212 base pair product) [20]. The PCR reactions were subjected to 1 cycle of 95 °C for 5 min, followed by 45 cycles of 95 °C for 30 s, 60 °C for 30 s, and 72 °C for 45 s. The beta actin gene [20] was used to normalize the relative amounts of mRNA expression of FcγRI gene for the same sample. The equation 2^− (ΔΔCt)^ was used to quantitate the relative expression levels [21]. Each sample was conducted in duplicate [11, 20, 22]. In addition, cDNA from each RNA extract was used for conventional RT-PCR to quality check the PCR product compared with those in previous studies [20, 21].

### Determination of FcγRI expression on monocytes using FACS

Overall, 2 × 10^6^ PBMC (CD27^−^) were cultured in RPMI1640 supplemented with 10 % of FBS alone or plus IL-2 (10 ng/ml), TGF-β1 (100 pg/ml), or ESA (0.1 μg/ml). After incubation for 12 h, cultured PBMC were washed in 0.1 M PBS containing 0.01 % of BSA and then stained with anti-FcγRI (CD64). The cells were then stained with anti-human CD14 to identify monocytes (CD14^+^). The intensity of expression was then analyzed with a FACSCalibur flow cytometer and CellQuest software (Becton Dickinson, CA, USA).

### Determination of phagocytic activity by AB serum opsonized zymosan phagocytosis assay

#### Prepared opsonized FITC-conjugated ZM

Serum opsonization of ZM provided optimal adhesion and phagocytosis. Briefly, 30 mg of ZM A conjugated to FITC was incubated with 2 ml of heat-inactivated human AB serum for 45 min at 37 °C. After washing with PBS, opsonized ZM was suspended in 0.1 % albumin and maintained at −40 °C until use (modified from [23]).

#### Measurement of the ESA effect on FcγRI-mediated phagocytosis

We conducted an opsonized ZM phagocytosis assay on monocytes in a PBMC culture (modified from [24]). Briefly, 3 × 10^6^ PBMC (CD27^−^) were cultured in RPMI 1640 supplemented with 10 % FBS, either alone or with the addition of IL-2 or ESA (0.1 μg/ml). After 12 h of incubation, the non-adherent cells were removed. The adherent cells were gently washed three times with RPMI. An approximate ratio of opsonized FITC-conjugated ZM/adherent cells of 10:1 was incubated for 1.5 h at 37 °C in complete media, then washed three times in PBS. Excess ZM particles were removed by 100 U/ml of lyticase for 10 min at RT. After washing with PBS twice, the adherent cells were fixed with 1 % paraformaldehyde in 0.1 M PBS for 5 min and then scraped gently. The adherent cells were labeled with PE-conjugated anti-human CD14 to identify monocytes. The percentage of phagocytic cells (ZM^+^CD14^+^ cells) in total CD14^+^ cells was counted by FACS. Phagocytic capacity that was determined by the number of ZM particles internalized by phagocytic cells per 100 monocytes (CD14^+^) [25] were counted with a BX 41 (Olympus, Shinjuku-ku, Tokyo, Japan) fluorescent microscope and a FV10i-DOC (Olympus) confocal fluorescent microscope.

### Statistical analysis

Statistical analyses were performed by Student *t* test and analysis of variance (ANOVA) using the Statistical Package for the Social Science (SPSS) version 11.5 (SPSS Inc., Chicago, IL, USA). The results were considered statistically significant (*p* < 0.05) at the 95 % confidence interval.

## Results

### Low-dose *G. spinigerum* ESA (0.1 μg/ml) was appropriate for PBMC culture

**Fig. 1:**
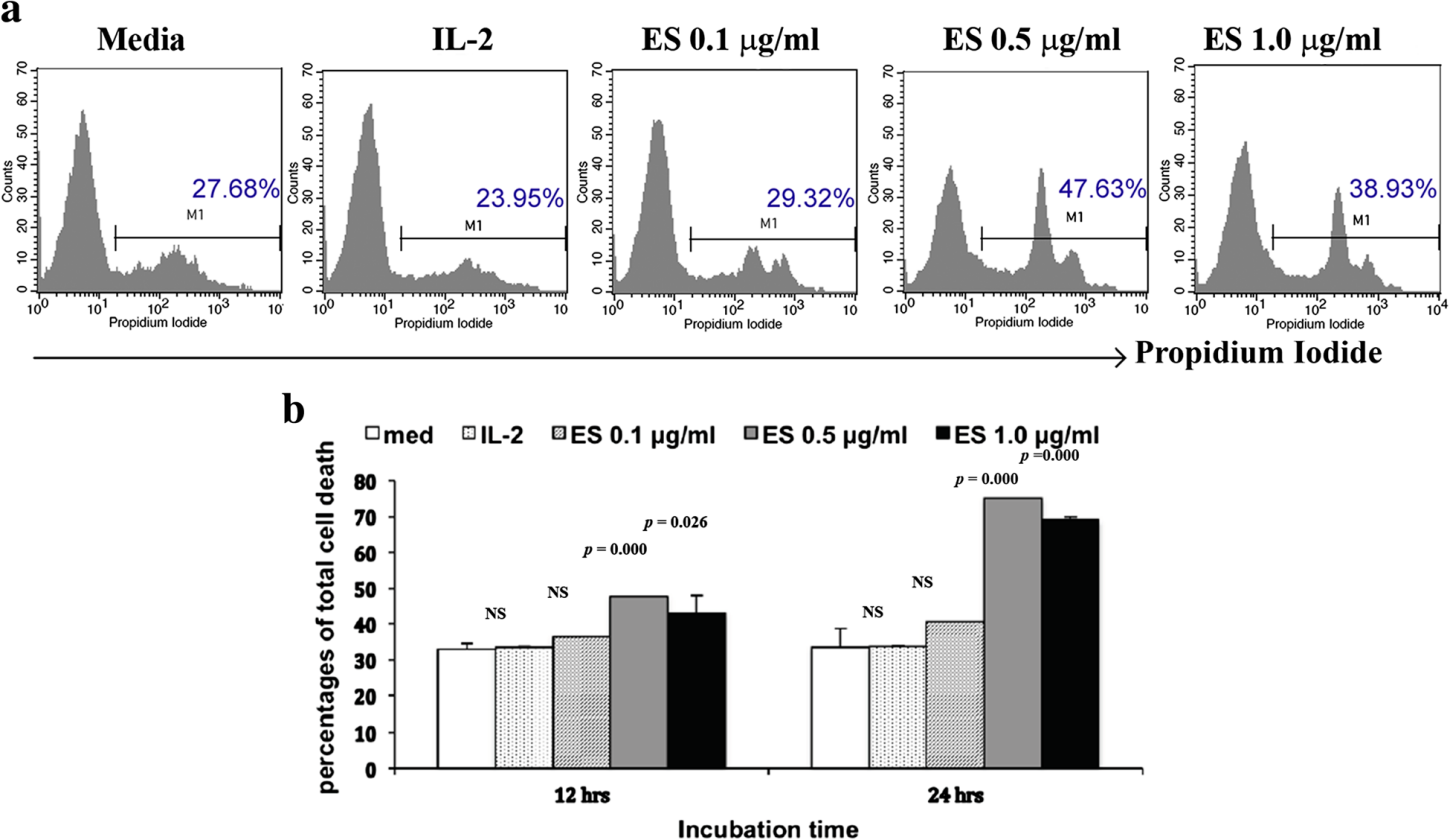
Determination of the appropriate *G. spinigerum* ESA concentration for PBMC culture; 2 million PBMC (CD27^−^) were cultured in RPMI 1640 supplemented with 10 % of inactivated FBS alone or plus IL-2 (10 ng/ml) or ESA (0.1, 0.5, and 1 μg/ml). After incubation for 12 or 24 h, the cultured PBMC were stained with PI. The intensity of PI-positive staining was then analyzed by FACSCalibur flow cytometer and CellQuest software (Becton Dickinson, San Jose, CA, USA). **a** Histograms compare the amount of dead cells (PI-positive staining cells) among PBMC treated with different conditions for 12 h. **b** Percentages of total dead cells in PBMC cultures compared among in medium alone, or each condition at 12 and 24 h of incubation. Each data element represents mean ± SEM from three independent experiments using three buffy coats. Significant differences among groups are indicated; *NS* not significant. The phenotype of PBMC treated with ESA (0.1 μg/ml) was similar to those in medium alone or plus IL-2 for 24 h. Therefore, the ESA at the dose of 0.1 μg/ml was the optimal condition for PBMC culture in this study

Cultures of normal PBMC were used as a human immune cell model. FACS and PI staining provides a rapid and reliable method to quantify viable cells in a cell suspension. Fig. 1 shows significant induction of apoptosis and possibly toxicity on PBMC, while the figure shows a comparable proportion of cell death from 0.1 μg/ml ESA as compared to medium alone, the basis of which 0.1 μg/ml was selected as appropriate for the experiments. The FACS data (image) show significant G2/M arrest as soon as ESA was added, as seen in the second G2 peak that emerged in the PI staining. A phenotype or histogram similar to medium alone or IL-2 histogram would be an appropriate condition for the assay. However, 0.1 μg/ml, although showing signs of G2/M arrest, may be acceptable, given that the effect at a higher concentration was less pronounced. Moreover, statistical analysis revealed that the numbers of necrotic PBMC cultured in medium alone, with IL-2, or ESA (0.1 μg/ml) were not significantly different (*p* > 0.05) during 24 h of incubation. Therefore, *G. spinigerum* ESA at a dose of 0.1 μg/ml was optimal for PBMC culture.

### Effect of *G. spinigerum* ESA on the transcription of FcγRI mRNA in monocytes of PBMC culture

**Fig. 2:**
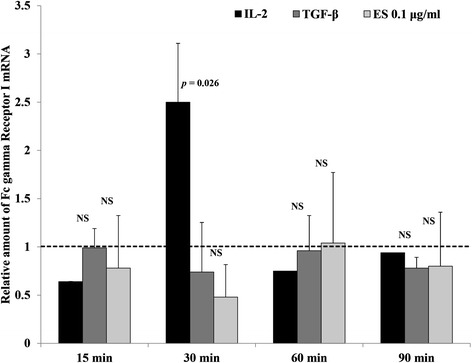
Effect of *G. spinigerum* ESA on FcγRI mRNA expression in monocytes. Approximately 3 × 10^6^ PBMC (CD27^−^) were cultured in complete medium alone or complete medium plus IL-2 (10 ng/ml), TGF-β1 (100 pg/ml), or ESA (0.1 μg/ml) for 15–90 min. After incubation, the cultured PBMC were harvested and processed to extract total RNA. For each culture, qRT-PCR using cDNA synthesized from 1 μg of total RNA template was performed in duplicate using specific primers (1 μM) for FcγRI and β-actin in a LightCycler 480 instrument. For each sample, the β-actin gene mRNA was used to normalize the relative amounts of mRNA expression for the FcγRI genes. Results are expressed as fold change relative to PBMC in medium alone. The data represent mean ± SEM from three independent experiments using three buffy coats. Significant differences among groups are indicated; *NS* not significant. Results are *G. spinigerum* ESA (0.1 μg/ml) tended to slightly down-regulate FcγRI mRNA expression compared with medium alone (*p* > 0.05)

FcγRI mRNA expression in monocytes of PBMC culture was measured by qRT-PCR during 90 min of cultivation. The results were expressed as twofold increase relative to PBMC cultured in medium alone. We found that *G. spinigerum* ESA (0.1 μg/ml) tended to down-regulate FcγRI mRNA expression compared with medium alone (Fig. 2).

**Fig. 3:**
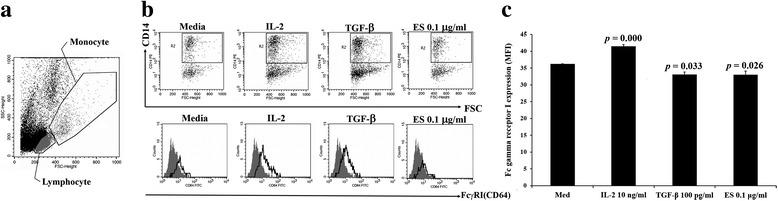
*G. spinigerum* ESA modulated the expression of FcγRI on monocytes. Approximately 2 × 10^6^ PBMC (CD27^−^) were cultured in medium alone or in medium plus IL-2 (10 ng/ml), TGF-β1 (100 pg/ml), or ESA (0.1 μg/ml) for 12 h. After incubation, the cultured PBMC were stained with anti-FcγRI (CD64) and anti-human CD14 to identify monocyte (CD14^+^). The intensity of FcγRI expression was then analyzed by FACSCalibur flow cytometer with CellQuest software (Becton Dickinson, San Jose, CA, USA). **a** Monocytes and lymphocytes in PBMC cultures were gated based on their forward/sideward scatter characteristic. **b** FACS *dot plots* (*upper panel*) and FACS histograms (*lower panel*) show the expression of FcγRI on monocytes (CD14^+^CD64^+^). **c** Comparison of FcγRI expression (mean of fluorescence intensity; *MFI*) on monocytes in PBMC culture in medium alone, plus IL-2, TGF-β, or ES 0.1 μg/ml. In each FACS histogram, the *grey* profiles represent isotype control and the *white* profiles represent the amount of FcγRI expression. The results presented are from one experiment and are representative of data from three to four independent experiments. Significant differences among groups are indicated; *NS* not significant. ESA (0.1 μg/ml) significantly decrease FcγRI phenotypic expression on monocytes at 12 h of cultivation in comparison to those in medium alone (*p* = 0.026). The PBMC cultured with IL-2 and TGF-β show appropriated significant increased and decreased FcγRI expression, respectively, in comparing to the control

FACS analysis showed that ESA (0.1 μg/ml) significantly decreased FcγRI phenotypic expression on monocytes at 12 h of cultivation in comparison to monocytes in medium alone (*p* = 0.026) (Fig. 3).

In each experiment, we included PBMC cultured with IL-2 [15] and TGF-β1 to compare up- and down-transcription of FcγRI and expression, respectively, with those in medium alone or plus ESA.

### Impaired phagocytosis of ESA-treated monocytes

**Fig. 4:**
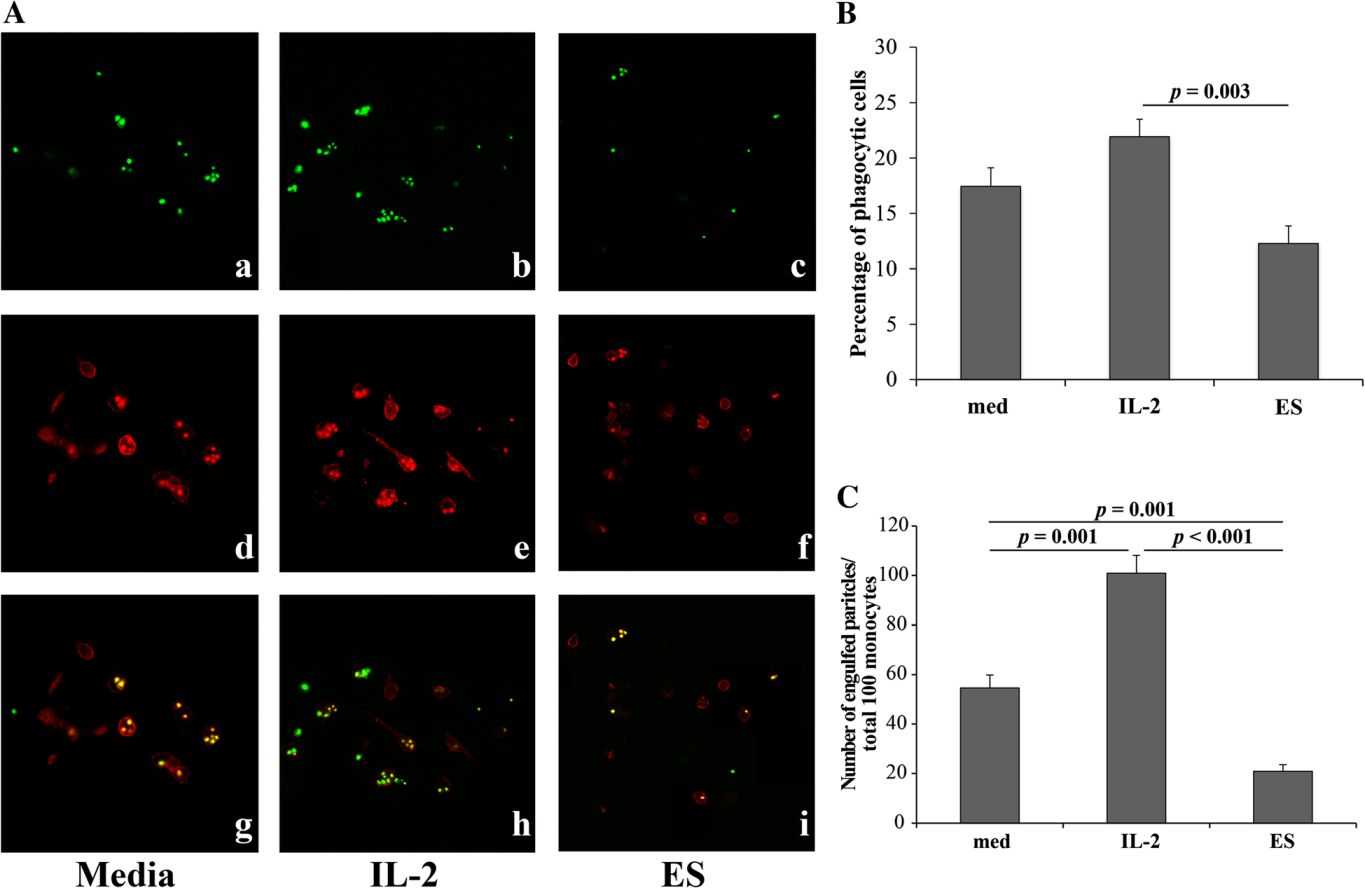
*G. spinigerum* ESA modulated phagocytic capacity of monocytes. Monocyte-enriched adherent cells from PBMC cultured in medium alone, plus IL-2 (10 ng/ml) or ES (0.1 μg/ml) for 12 h were incubated with zymosan (ZM). After cultivation, the cells were labeled with anti-human CD14 PE to identify monocytes and assayed phagocytosis by confocal microscope (**A**). ZM particle in phagocytic cells were shown in *green* (*a*–*c*), total monocytes were shown in *red* (*d*–*f*), and merged micrographs (*g*–*i*) illustrated total monocytes with and without engulfed ZM. The micrographs represent one of three independent experiments. (**A**) The percentage of phagocytic cells (ZM^+^CD14^+^ cells) in total CD14^+^ cells by FACSCalibur. (**B**) The phagocytic capacity were determined from the number of ZM particles in phagocytic cells of total 100 monocytes (CD14^+^) and were counted under fluorescent microscope and confocal microscope (**C**). Each data element represents mean ± SEM from three independent experiments. The difference in value between groups was analyzed by Student’s *t* test. Significant differences among groups are indicated; *NS* not significant. The results are the number of internalized ZM in 100 monocytes in ESA-treated PBMC cultures was significantly lower than those in medium alone (*p* = 0.001). The number of phagocytic cells (percentages of monocytes with engulfed ZM) in ESA-pretreated culture tended to be lower than controls cultured in medium alone (*p* > 0.05). IL-2 pretreatment significantly increased the number of phagocytic cells (*p* = 0.003) and phagocytosis capacity (*p* < 0.001) compared with ESA-pretreated cells

We performed a phagocytosis assay of serum-opsonized ZM particles in monocytes to measure their phagocytic capacity. FITC-labeled ZM particles were used to test the functional consequences of the particles binding to FcγRI. The numbers of internalized ZM in 100 monocytes were used to determine phagocytic capacity. The monocytes in ESA-treated PBMC cultures had a significantly decreased phagocytotic capacity (number of ZM/100 monocytes). We found that the number of internalized ZM per 100 monocytes in ESA-treated PBMC cultures was significantly lower than those in medium alone (*p* = 0.001). The number of phagocytic cells (percentage of monocytes with engulfed ZM) in ESA-pretreated culture tended to be lower than controls cultured in medium alone (*p* > 0.05). IL-2 pretreatment significantly increased the number of phagocytic cells (*p* = 0.003) and phagocytosis capacity (*p* < 0.001) compared with ESA-pretreated cells (Fig. 4).

## Discussion

The current study is the first to demonstrate that *G. spinigerum* L3 ESA modulates FcγRI-mediated monocyte functions in less than 12 h. Using the PBMC model, we demonstrated that 0.1 μg/ml of ESA could significantly decrease FcγRI expression on the monocyte surface. We also found a significant decrease in the FcγRI-mediated phagocytotic capacity of monocytes in response to ESA pretreated-PBMC culture although the percentages of phagocytic cells were not significantly depleted.

Importantly, ESA at a dose of 0.1 μg/ml in PBMC culture during 24 h of observation still caused signs of G2/M arrest. This evidence was less pronounced as the concentration was reduced (Fig. 1). Therefore, it would be interesting to observe the incubation under concentrations of ESA <0.1 μg/ml in further studies.

Previous studies reported that ES protein molecules from helminths are influential, particularly for larval migration in tissues. ES proteins have divergent functions and are composed of various protein molecules and enzymes that reduce the viability of host immune cells via direct cytolysis [3–5] or by promoting apoptosis [26–28]. A previous study of PBMC co-culture with live *Brugia malayi* L3 revealed a decrease in total cell numbers at 72 h cultivation time by apoptosis [26]. Consistently, we observed increased cell death during 24 h of incubation with high concentration (0.5–1.0 μg/ml) of *G. spinigerum* ESA (Fig. 1).

The present study aimed to explain how *G. spinigerum* L3 ESA is involved in interfering with the role of monocytes in the host immune response. In this study, we could not address whether ESA could change the FcγRI configuration, which might contribute to the depletion of phagocytosis capacity. We suggest that decreased expression of FcγRI was probably caused by the down-regulation of its transcription. However, our results were unable to confirm this hypothesis. We also assume that the change subsequently interfered with the initiation of FcγRI signaling cytolysis of monocyte phagocytosis. The first step of monocyte-mediated phagocytosis is the adherence of particles or foreign bodies to the monocyte membrane by FcγRI. Then, FcγRI crosslinking leads to γ-chain tyrosine phosphorylation that interacts with p72syk protein kinase. This is essential for FcγRI-mediated phagocytosis [7]. These receptors undergo rearrangements of the actin skeleton leading to internalization of the particles, vacuole formation, and induction of inflammatory responses [29]. In this study, we used AB+ serum-opsonized ZM as a particle for the phagocytosis assay. This assay is commonly used to determine phagocytic capacity [30]. We found significantly diminished phagocytic activity in PBMC pretreated with ESA (Fig. 4). Therefore, we demonstrated that ESA-impaired FcγRI-mediated phagocytosis.

The mechanisms by which ESA affects host immunity are complicated, diverse, and vary among different types of helminths [1]. Previous studies suggested that dysregulated FcγRI expression might have dysfunctional consequences as activated FcγR has a pivotal role in phagocytosis, cytolysis, and the induction of inflammatory cytokines [7]. These ES products also have immune-modulatory effects on APCs such as DC in vitro and in vivo [18], particularly after repeated exposure, which can impact on downstream modulation of anti-schistosome responses and immunopathology in the liver [31]. Moreover, larva hookworms have been shown to activate macrophage to trap the larva migration [12].

FcγRI is also involved in inflammatory responses induced by C-reactive protein [32]. The 66-kDa form of ESA from *Haemonchus contortus* consistently inhibited monocyte function by decreasing the production of hydrogen peroxide and nitric oxide in vitro [33, 34]. Moreover, ES products from adult *Dirofilaria immitis* have been shown to down-regulate monocyte transmigration [35]. Similar to our study, monocytes exposed to *B. malayi* microfilariae (mf) or their ES showed significantly decreased ability to phagocytose opsonized *Escherichia coli* bioparticles [11].

FcγRI-mediated phagocytosis in helminthic infection is not the only mechanism to respond against infective larvae in patients. As reported in earlier studies, in addition to phagocytes, innate and adaptive immune responses such as antibody formation, production of pro-inflammatory cytokines and anti-inflammatory cytokines, and anti-organism peptides and proteins are involved. These interplays of immunologic responses to infection and the localization of migrating phagocytes act to defend an organism against invasion [12]. In addition, FcγRs regulate innate immune effector cell activation and are also involved in adaptive immunity by regulating specific antibody production [7].

In this study, we performed a phagocytosis assay to investigate potential decreases in FcγRI expression and bioactivity in monocytes. Similarly, monocyte dysfunction in filarial infection is one of the various mechanisms proposed to address the diminished parasite antigen-specific T-cell responses seen with patent lymphatic filariasis. Monocytes from filarial infection were laden with filarial antigen, exhibit diminished expression of genes involved in antigen presentation and processing, and produce fewer pro-inflammatory cytokines in response to surface receptor cross-linking. In addition, these monocytes had a lower expression of toll-like receptors (TLRs), leading to depleted cytokine production in response to TLR engagement [11].

In agreement with our findings (see Additional files 1, 2 and 3), the PBMC treated with ESA from live L3 for 18 h showed the profiles of 63 down-regulated genes, with functions involving cytotoxicity immunity, including gene groups of TLRs, IgG and IgE Fc receptors, granzymes in cytotoxic T cells, and the family of killer cell receptors (Table S1, in Additional file 2). Down-regulated gene profiling showed that genes involved in innate immunity had a role in the prolonged survival of the larva migrans [6].

This study had a number of limitations, which include the following: (1) microarray analysis (see Additional files 1, 2 and 3) was performed only once and with a limited time point. Therefore, the gene profiling of immune responses to the ESA may not be complete. However, the results were useful and contributed to our hypothesis. The additional experiment revealed the effect of ES released from the live *G. spinigerum* L3 in a non-contact system on PBMC culture. It proved that the findings in our study were caused by ES alone and not from direct larval attack. However, FACS analysis consistently showed significant down-regulation of FcγRI expression on monocytes. (2) Result of FcγRI mRNA expression was unclear due to incomplete experimental design including too short observation time, duplication of running qRT-PCR, and small sample size. In further study, to observe at least 24 h [36] and run qRT-PCR in triplicate will be more reliable [37, 38]. (3) It is not clear whether components in the ESA directly down-regulated FcγRI expression or had an indirect synergistic or antagonistic effect on the expression of pro-inflammatory and anti-inflammatory cytokines. Previous studies reported that IFN-γ [7] and IL-2 [9] induced FcγRI expression while TGF-β1 inhibited its expression [18]. Consistently, we found that the effect of ESA was to down-regulate mRNA expression of cytokines and receptors such as IFN-γ, IL-2R, IL-12R, and IL-18R (Table S1, see Additional file 2) and up-regulated IL-1, IL-1α, IL-19, IL-24, and TNF-R (Table S2, see Additional file 3). We did not investigate additional factors that might contribute to FcγRI signaling. For instance, phospholipase D, sphingosine kinase, and S1P activation can affect FcγRI signaling and consequent phagocytosis [20, 39].

Further studies with a larger sample size and lower dose of ESA are needed in further experiments to prove the current hypothesis and also to address other mechanisms that might contribute to the immune evasive strategies of *G. spinigerum* L3, such as defective NK cell function, the induction of immune cell apoptosis, and cytokine interplay. These findings will clarify the pathogenesis of human gnathostomiasis and hopefully aid in the development of an effective treatment.

## Conclusions

In conclusion, this study indicates that ESA may affect monocyte function by (1) down-regulating the transcription of critical genes, (2) decreasing phenotypic FcγRI expression, and (3) subsequently reducing biological phagocytic activity. Thus, impaired FcγRI-mediated phagocytosis might be one mechanism that allows *G. spinigerum* L3 to evade the immune system. This study provides preliminary information for future in-depth studies to clarify the complicated mechanisms of immune-evasive strategy of *G. spinigerum* larvae.

## Additional files

Additional file 1: Immune response-related gene profiling in PBMC induced by *G. spinigerum* excretory secretion (ES) from non-contact live third stage larvae (L3) in a Transwell co-culture system (DOCX 40 kb) Additional file 2: Table S1. The emphasized down-regulated genes in transcriptional profiles of PBMC induced by *G. spinigerum* ES from non-contact live L3 co-culture (DOC 79 kb) Additional file 3: Table S2. The emphasized up-regulated genes in transcriptional profiles of PBMC induced by *G. spinigerum* ES from non-contact live L3 co-culture (DOC 62 kb)
