# Supplementary material for: Third-stage Gnathostoma spinigerum larva excretory secretory antigens modulate function of Fc gamma receptor I-mediated monocytes in peripheral blood mononuclear cell culture
Source: Trop Med Health. 2016 Apr 21;44:5. doi: 10.1186/s41182-016-0005-x (PMC4934145; doi:10.1186/s41182-016-0005-x)
Supplement: Additional file 1: — Immune response-related gene profiling in PBMC induced by G. spinigerum excretory secretion (ES) from non-contact live third stage larvae (L3) in a Transwell co-culture system (DOCX 40 kb) [file 41182_2016_5_MOESM1_ESM.docx]

**Immune response-related gene profiling in PBMC induced by *G. spinigerum* excretory secretion (ES) from non-contact live third stage larvae (L3) in a Transwell co-culture system**

This study aimed to examine gene profiling correlated with immune responses on PBMC induced by ES released from non-contact live *G. spinigerum* L3 for 18 h using DNA microarray analysis.

**Preparation of peripheral blood mononuclear cells (PBMC)**

Buffy coats from O^+^ blood from healthy donors were purchased from the Thai Red Cross Society. Gradient centrifugation and Lymphoprep (Axis-Sheld Poc AS, Oslo, Norway) were used to separate PBMC from buffy coat samples as previously described [[1](#_ENREF_1)]. Adaptive immunity is comprised of immune cell interactions and components. To limit interference from adaptive immunity, anti-human CD27 and LD columns [[2](#_ENREF_2)] were used to remove memory cells (CD27^+^) from each PBMC sample. Based on FACSCalibur flow cytometry, PBMC used in the experiment comprised less than 1% of memory cells.

**PBMC co-cultured with non-contact live *G. spinigerum* L3**

After quiescent cultivation in medium composed of RPMI1640 + 1% inactivated fetal bovine serum (FBS) overnight, 5 × 10^6^ PBMC were seeded in a 6-well Transwell cell culture (Corning, NY, USA) and 5 live *G. spinigerum* L3 (modified from [[3](#_ENREF_3)],[[4](#_ENREF_4)]) were seeded in the top well of the PBMC cultures [[5](#_ENREF_5)] for 18 h [[6](#_ENREF_6)].

**Determination of gene profiling of the stimulated PBMC using DNA microarray analysis**

At 18 h cultivation the PBMC were then harvested and washed in 0.1 M PBS. Total RNA was extracted from the stimulated PBMC and control PBMC (in complete medium alone) (n=3) using the RNA isolation kit and DNase treatment step (Qiagen, Hilden, Germany) and DNase treatment step. Total RNA was quantified using a NanoDrop ND-1000 spectrophotometer with ND-1000 3.3 software, and RNA integrity (RIN) was examined using an Agilent Bioanalyzer. Affymetrix GeneChip® Human Gene 1.0 ST. Arrays were performed on 5 μg of total RNA, according to a standard protocol from the manufacturer (Affymetrix Inc., Santa Clara, CA, USA). The data were analyzed by Agilent GeneSpring GX Software version 12.0 (License No. NQO9-4661-4168). Differential expression genes were identified using the criteria of a > 1.5-fold increase/decrease in gene expression in the treated group in comparison to those cultured in medium alone [[7](#_ENREF_7)].

**Results and Discussion**

During 18 h of cultivation, PBMC were exposed to ES released from live L3 in a non-contact culture. Therefore, only ES caused the changes in PBMC but not the larvae directly attacked. These PBMC exhibited 107 genes that were significantly changed compared to normal PBMC (at fold change ≥1.5, *P*-value < 0.05). These included 63 down-regulated and 44 up-regulated genes (Tables S1 and S2).

Table S1 shows the profiles of 63 down-regulated genes, with functions involving cytotoxicity immunity, including gene groups of Toll-like receptors, IgG and IgE Fc receptors, granzymes in cytotoxic T cells, and the family of killer cell receptors. Down-regulated gene profiling showed genes involved in innate immunity had a role in the prolonged survival of the larva migrans [[8](#_ENREF_8)]

Using a non-contact co-culture system, gene profiles of cultured PBMC were analyzed to explain the human immune response to the ES secreted during Gnathostomiasis alone without direct larval attack. The incubation time was chosen based on an earlier study that investigated the innate immune response of IFN-γ mediated gene profiling in naïve macrophages induced by *Mycobacterium tuberculosis* exosomes [[6](#_ENREF_6)]. The use of 18 h incubation might not be the optimal time point to observe marked differential expression of mRNA. Therefore, the differential expression of both receptors investigated in this study were slightly above the significant 1.5-fold change (*P* < 0.05 (range, −2.903 to −2.013) (Table S1). Here, we performed only one experiment in triplicate using one donor. However, the down-regulated gene profiling was reasonable. The results were consistent with our findings in PBMC cultured with ESA prepared from *G. spinigerum* L3 culture (the main manuscript) including decreased FcγRI expression on monocytes (by FACS analysis after 12 h incubation), and down transcriptional regulation of FcγRI mRNA (by qRT-PCR during 90 min incubation)

We found ES from the live L3 down-regulated the mRNA expression of cytokines and receptors such as IFN-γ, IL-2R, IL-12R, and IL-18R (Table S1), and up-regulated IL-1, IL-1αlpha IL-19, IL-24, and TNF-R (Table S1). Moreover, Table S2 shows some apoptosis related gene profiles significantly upregulated in the PBMC exposed to the live *G. spinigerum* L3 ES. Our further study should be conducted to elucidate roles of the *G. spinigerum* ES or ESA from the larva culture in apoptosis and cytokine productions of host immune cells.

**References**

1. Boyum A. Isolation of mononuclear cells and granulocytes from human blood. Isolation of monuclear cells by one centrifugation, and of granulocytes by combining centrifugation and sedimentation at 1 g. Scandinavian journal of clinical and laboratory investigation Supplementum. 1968;97:77-89.

2. Somthana K, Eshita Y, Kumsiri R, Dekumyoy P, Waikagul J, Kalambaheti T et al. Roles of partially purified antigens from Gnathostoma spinigerum larvae on antibody production by human B cell culture. The Southeast Asian journal of tropical medicine and public health. 2011;42(4):772-81.

3. Saksirisampant W, Thaisom S, Ratanavararak M, Thanomsub BW. Gnathostoma spinigerum: immunodepression in experimental infected mice. Experimental parasitology. 2012;132(3):320-6. doi:10.1016/j.exppara.2012.08.002.

4. Maleewong W, Wongkham C, Intapan P, Pariyanonda S, Morakote N. Excretory-secretory antigenic components of Paragonimus heterotremus recognized by infected human sera. Journal of clinical microbiology. 1992;30(8):2077-9.

5. Scholzen A, Mittag D, Rogerson SJ, Cooke BM, Plebanski M. Plasmodium falciparum-mediated induction of human CD25Foxp3 CD4 T cells is independent of direct TCR stimulation and requires IL-2, IL-10 and TGFbeta. PLoS pathogens. 2009;5(8):e1000543. doi:10.1371/journal.ppat.1000543.

6. Singh PP, LeMaire C, Tan JC, Zeng E, Schorey JS. Exosomes released from M. tuberculosis infected cells can suppress IFN-gamma mediated activation of naive macrophages. PloS one. 2011;6(4):e18564. doi:10.1371/journal.pone.0018564.

7. Mavrommatis B, Offord V, Patterson R, Watson M, Kanellos T, Steinbach F et al. Global gene expression profiling of myeloid immune cell subsets in response to in vitro challenge with porcine circovirus 2b. PloS one. 2014;9(3):e91081. doi:10.1371/journal.pone.0091081.

8. Moore DA, McCroddan J, Dekumyoy P, Chiodini PL. Gnathostomiasis: an emerging imported disease. Emerging infectious diseases. 2003;9(6):647-50. doi:10.3201/eid0906.020625.
