# Supplementary material for: Third-stage Gnathostoma spinigerum larva excretory secretory antigens modulate function of Fc gamma receptor I-mediated monocytes in peripheral blood mononuclear cell culture
Source: Trop Med Health. 2016 Apr 21;44:5. doi: 10.1186/s41182-016-0005-x (PMC4934145; doi:10.1186/s41182-016-0005-x)
Supplement: Additional file 2: Table S1. — The emphasized down-regulated genes in transcriptional profiles of PBMC induced by G. spinigerum ES from non-contact live L3 co-culture (DOC 79 kb) [file 41182_2016_5_MOESM2_ESM.doc]

Table S1 The emphasized downregulated genes in transcriptional profiles of PBMC induced by *G. spinigerum* ES from non-contact live L3 co culture.

Sixty-three immunity-related genes were downregulated in PBMC during an 18-h treatment. Downregulation was defined as a ≥1.5-fold reduction in transcript levels relative to PBMC cultured in medium alone (*p*-value ≤ 0.05).

^a^ denotes genes encoding receptors in the killer cell lectin-like receptor subfamily,

^b^ denotes genes encoding Fc fragment of IgG, high affinity Ia, receptors (CD64 or FcγRI)

| **Transcripts Cluster ID** | **Gene symbol** | **Gene description** | **Fold change** | **GenBank** |
| --- | --- | --- | --- | --- |
| 7906443 | FCER1A | Fc fragment of IgE, high affinity I, receptor for; alpha polypeptide | -3.356 | X06948 |
| 8151101 | MYBL1 | v-myb myeloblastosis viral oncogene homolog (avian)-like 1 | -3.199 | BC101186 |
| 8094533 | FLJ16686 |  | -3.179 | AK131492 |
| 7988672 | HDC | histidine decarboxylase | -3.035 | BC130527 |
| 7961175^a^ | KLRC3 | killer cell lectin-like receptor subfamily C, member 3 | -2.903 | L14542 |
| 8105331 | GZMK | granzyme K (granzyme 3; tryptase II) | -2.781 | BC035802 |
| 7961166^a^ | KLRC4|KLRK1 | killer cell lectin-like receptor subfamily C, member 4 | killer cell lectin-like receptor subfamily K, member 1 | -2.766 | AJ001683 |
| 7905047^b^ | FCGR1A|FCGR1B | Fc fragment of IgG, high affinity Ia, receptor (CD64) | Fc fragment of IgG, high affinity Ib, receptor (CD64) | -2.750 | BC152383|L03419 |
| 8044049 | IL18RAP | interleukin 18 receptor accessory protein | -2.745 | AF077346 |
| 8049297 | SCARNA5 | small Cajal body-specific RNA 5 | -2.698 |  |
| 7934979 | ANKRD1 | ankyrin repeat domain 1 (cardiac muscle) | -2.638 | BC018667 |
| 7902205 | IL12RB2 | interleukin 12 receptor, beta 2 | -2.557 | U64198 |
| 8105340 | GZMA | granzyme A (granzyme 1, cytotoxic T-lymphocyte-associated serine esterase 3) | -2.541 | M18737 |
| 7934898 | ANKRD22 | ankyrin repeat domain 22 | -2.533 | BC021671 |
| 7953737 | CLEC6A | C-type lectin domain family 6, member A | -2.519 | AY321309 |
| 7963575 | EIF4B | eukaryotic translation initiation factor 4B | -2.406 |  |
| 8143471 | CLEC5A | C-type lectin domain family 5, member A | -2.398 | AK292766 |
| 7961182^a^ | KLRC2|KLRC3 | killer cell lectin-like receptor subfamily C, member 2 | killer cell lectin-like receptor subfamily C, member 3 | -2.392 | BC093644|AF461157|AF350017 |
| 7905060^b^ | FCGR1A|FCGR1B | Fc fragment of IgG, high affinity Ia, receptor (CD64) | Fc fragment of IgG, high affinity Ib, receptor (CD64) | -2.384 | BC032634|L03420 |
| 7981068 | SERPINA1 | serpin peptidase inhibitor, clade A (alpha-1 antiproteinase, antitrypsin), member 1 | -2.370 | BC011991 |
| 7919133^b^ | FCGR1A|FCGR1B | Fc fragment of IgG, high affinity Ia, receptor (CD64) | Fc fragment of IgG, high affinity Ib, receptor (CD64) | -2.358 | L03419|BC032634 |
| 7964787 | IFNG | interferon, gamma | -2.342 | BC070256 |
| 7981988 | SNRPN|SNORD116-20|SNORD116@ | small nuclear ribonucleoprotein polypeptide N | small nucleolar RNA, C/D box 116-20 | small nucleolar RNA, C/D box 116 cluster | -2.330 | AF241255 |
| 7953749 | CLEC4D | C-type lectin domain family 4, member D | -2.240 | AY115592 |
| 7981996 | SNRPN|SNORD116-24 | small nuclear ribonucleoprotein polypeptide N | small nucleolar RNA, C/D box 116-24 | -2.233 |  |
| 8096919 | ALPK1 | alpha-kinase 1 | -2.217 | BC060780 |
| 7920244 | S100A8 | S100 calcium binding protein A8 | -2.210 | AK291328 |
| 7977507 | RPPH1 | ribonuclease P RNA component H1 | -2.204 |  |
| 7926410 | MRC1|MRC1L1 | mannose receptor, C type 1 | mannose receptor, C type 1-like 1 | -2.182 | J05550|BC142642 |
| 7926451 | MRC1|MRC1L1 | mannose receptor, C type 1 | mannose receptor, C type 1-like 1 | -2.182 | J05550|BC142642 |
| 8014316 | CCL5 | chemokine (C-C motif) ligand 5 | -2.180 | M21121 |
| 8030362 | SNORD33|RPL13A | small nucleolar RNA, C/D box 33 | ribosomal protein L13a | -2.163 | AK056837 |
| 7961059^a^ | KLRB1 | killer cell lectin-like receptor subfamily B, member 1 | -2.163 | AK292022 |
| 8011415 | P2RX5 | purinergic receptor P2X, ligand-gated ion channel, 5 | -2.147 | BC039015 |
| 8049299 | SCARNA6 | small Cajal body-specific RNA 6 | -2.144 |  |
| 8083260 | CPA3 | carboxypeptidase A3 (mast cell) | -2.140 | BC012613 |
| 7905571 | S100A9 | S100 calcium binding protein A9 | -2.138 | M26311 |

| **Transcripts Cluster Id** | **Gene symbol** | **Gene description** | **Fold change** | **Genbank** |
| --- | --- | --- | --- | --- |
| 7961187^a^ | KLRC1 | killer cell lectin-like receptor subfamily C, member 1 | -2.107 | BC012550 |
| 8139100 | TRGC2|TRG@|TARP | T cell receptor gamma constant 2 | T cell receptor gamma locus | TCR gamma alternate reading frame protein | -2.102 | BC039116|X06774|Y00790|X06776 |
| 7921275 | FCRL3 | Fc receptor-like 3 | -2.100 | AF416904 |
| 8115076 | CSF1R | colony stimulating factor 1 receptor, formerly McDonough feline sarcoma viral (v-fms) oncogene homolog | -2.088 | X03663 |
| 7942594 | SNORD15B | small nucleolar RNA, C/D box 15B | -2.078 |  |
| 7951535 | KDELC2 | KDEL (Lys-Asp-Glu-Leu) containing 2 | -2.071 | AY358616 |
| 8043236 | GNLY | granulysin | -2.064 | BC063245 |
| 7995783 | MT2A | metallothionein 2A | -2.053 | BC007034 |
| 7981978 | SNRPN|SNORD116-15 | small nuclear ribonucleoprotein polypeptide N | small nucleolar RNA, C/D box 116-15 | -2.043 |  |
| 7949410 | MALAT1 | metastasis associated lung adenocarcinoma transcript 1 (non-protein coding) | -2.040 |  |
| 8117458 | BTN3A1 | butyrophilin, subfamily 3, member A1 | -2.035 | AK290193 |
| 8168531 | GPR174 | G protein-coupled receptor 174 | -2.031 | BC104922 |
| 7961151^a^ | KLRK1|KLRC4 | killer cell lectin-like receptor subfamily K, member 1 | killer cell lectin-like receptor subfamily C, member 4 | -2.018 | AK292059|BC017784 |
| 7953892^a^ | KLRF1 | killer cell lectin-like receptor subfamily F, member 1 | -2.013 | AJ305370 |
| 8149399 | LONRF1 | LON peptidase N-terminal domain and ring finger 1 | -2.007 | BC106042 |
| 7931914 | IL2RA | interleukin 2 receptor, alpha | -1.746 | X01057 |
| 7934161 | PRF1 | perforin 1 (pore forming protein) | -1.677 | BC063043 |
| 7950578 | PAK1 | p21/Cdc42/Rac1-activated kinase 1 (STE20 homolog, yeast) | -1.797 | AK293098 |
| 7964787 | IFNG | interferon, gamma | -2.342 | BC070256 |
| 7978366 | GZMB | granzyme B (granzyme 2, cytotoxic T-lymphocyte-associated serine esterase 1) | -1.716 | AY372494 |
| 8011884 | FCER1A | NLR family, pyrin domain containing 1 | -1.647 | AF310105 |
| 8105340 | MYBL1 | granzyme A (granzyme 1, cytotoxic T-lymphocyte-associated serine esterase 3) | -2.541 | M18737 |
| 8129804 | FLJ16686 | mitogen-activated protein kinase kinase kinase 5 | -1.802 | BC088829 |
| 8143307 | HDC | homeodomain interacting protein kinase 2 | -1.589 | AF208291 |
| 8154178 | KLRC3 | Janus kinase 2 (a protein tyrosine kinase) | -1.786 | AF058925 |
| 8175393 | GZMK | Rac/Cdc42 guanine nucleotide exchange factor (GEF) 6 | -1.537 | BC039856 |
