# Supplementary material for: Third-stage Gnathostoma spinigerum larva excretory secretory antigens modulate function of Fc gamma receptor I-mediated monocytes in peripheral blood mononuclear cell culture
Source: Trop Med Health. 2016 Apr 21;44:5. doi: 10.1186/s41182-016-0005-x (PMC4934145; doi:10.1186/s41182-016-0005-x)
Supplement: Additional file 3: Table S2. — The emphasized up-regulated genes in transcriptional profiles of PBMC induced by G. spinigerum ES from non-contact live L3 co-culture (DOC 62 kb) [file 41182_2016_5_MOESM3_ESM.doc]

Table S2. The emphasized upregulated genes in transcriptional profiles of PBMC induced by *G.* *spinigerum* ES from non-contact live L3 co culture.

Forty-four immunity-related genes were upregulated in PBMC during an 18-h treatment. Upregulation was defined as a ≥1.5-fold increase in transcript levels relative to PBMC cultured in medium alone (*p*-value ≤ 0.05).

^a^ denotes genes involved in regulation of apoptosis.

| **Transcripts Cluster ID** | **Gene symbol** | **Gene description** | **Fold change** | **GenBank** |
| --- | --- | --- | --- | --- |
| 8114964 | SPINK1 | serine peptidase inhibitor, Kazal type 1 | 5.830 | M11949 |
| 8048864 | CCL20 | chemokine (C-C motif) ligand 20 | 4.492 | BC020698 |
| 7909271^a^ | IL 24 | interleukin 24 | 4.037 | AY641441 |
| 8156043 | PSAT1 | phosphoserine aminotransferase 1 | 3.403 | BC004863 |
| 8094778 | UCHL1 | ubiquitin carboxyl-terminal esterase L1 (ubiquitin thiolesterase) | 3.047 | BC000332 |
| 8101126 | CXCL10 | chemokine (C-X-C motif) ligand 10 | 2.828 | BC010954 |
| 8124492 | HIST1H2BK | histone cluster 1, H2bk | 2.594 | BC108737 |
| 8054712^a^ | IL1A | interleukin 1, alpha | 2.523 | BC013142 |
| 7948910 | SNORD25|SNHG1 | small nucleolar RNA, C/D box 25 | small nucleolar RNA host gene (non-protein coding) 1 | 2.502 | AK095849 |
| 8033257 | C3 | complement component 3 | 2.477 | BC150179 |
| 8045688 | TNFAIP6 | tumor necrosis factor, alpha-induced protein 6 | 2.472 | BC030205 |
| 8005475 | TRIM16L | tripartite motif-containing 16-like | tripartite motif-containing 16 | 2.445 | DQ232882|AK056026 |
| 8059565 | PID1 | phosphotyrosine interaction domain containing 1 | 2.436 | BC040164 |
| 7904433 | PHGDH | phosphoglycerate dehydrogenase | 2.392 | AF171237 |
| 7943715 | ZC3H12C | zinc finger CCCH-type containing 12C | 2.389 | AB096241 |
| 8040080 | RSAD2 | radical S-adenosyl methionine domain containing 2 | 2.339 | AF442151 |
| 7972548 | GPR18 | G protein-coupled receptor 18 | 2.326 | BC066927 |
| 7953603 | C1S | complement component 1, s subcomponent | 2.298 | BC056903 |
| 7983910 | AQP9 | aquaporin 9 | 2.243 | AF016495 |
| 7957850 | GAS2L3 | growth arrest-specific 2 like 3 | 2.236 | BC043366 |
| 7919787 | HORMAD1 | HORMA domain containing 1 | 2.196 | AY626344|CR533505 |
| 7929065 | IFIT1 | interferon-induced protein with tetratricopeptide repeats 1 | 2.194 | BC007091 |

| **Transcripts Cluster ID** | **Gene symbol** | **Gene description** | **Fold change** | **GenBank** |
| --- | --- | --- | --- | --- |
| 8102594 | TNIP3 | TNFAIP3 interacting protein 3 | 2.163 | AF277289 |
| 8048717 | SGPP2 | sphingosine-1-phosphate phosphotase 2 | 2.131 | AF542512 |
| 7977270 | LOC388022 | hypothetical gene supported by AK131040 | 2.118 | AK131040 |
| 7968883 | C13orf31 | chromosome 13 open reading frame 31 | 2.107 | BC035749 |
| 8131844 | GPNMB | glycoprotein (transmembrane) nmb | 2.074 | BC032783 |
| 8105481 | C5orf29 | chromosome 5 open reading frame 29 | 2.058 | BC063534|AK090960 |
| 7969482 | IRG1 | immunoresponsive 1 homolog (mouse) | 2.044 |  |
| 8012951 | CDRT1|TRIM16 | CMT1A duplicated region transcript 1 | tripartite motif-containing 16 | 2.029 | AB209899 |
| 7922418 | SNORD74 | small nucleolar RNA, C/D box 74 | 2.028 |  |
| 8044541 | IL1F9 | interleukin 1 family, member 9 | 2.011 | AF206696 |
| 7909250^a^ | IL19 | interleukin 19 | 1.901 | AY040367 |
| 7909271^a^ | IL24 | interleukin 24 | 4.037 | AY641441 |
| 7915160^a^ | RRAGC | Ras-related GTP binding C | 1.743 | AF323609 |
| 7932964^a^ | C1D | nuclear DNA-binding protein | 1.688 | BC005235 |
| 7958019^a^ | DRAM | damage-regulated autophagy modulator | 1.573 | BC018435 |
| 7970317^a^ | TFDP1 | transcription factor Dp-1 | 1.583 | BC011685 |
| 7977775^a^ | DAD1 | defender against cell death 1 | 1.542 | BC009798 |
| 8052698^a^ | C1D | nuclear DNA-binding protein | 1.633 | BC005235 |
| 8054712^a^ | IL1A | interleukin 1, alpha | 2.523 | BC013142 |
| 8075316^a^ | OSM | oncostatin M | 1.813 | BC011589 |
| 8114612^a^ | CD14 |  | 1.589 | BC010507 |
| 8149733^a^ | TNFRSF10B | tumor necrosis factor receptor superfamily, member 10b | 1.618 | AF016266 |
